# Supplementary material for: The origin and degassing history of the Earth's atmosphere revealed by Archean xenon
Source: Nat Commun. 2017 May 18;8:15455. doi: 10.1038/ncomms15455 (PMC5454381; doi:10.1038/ncomms15455)
Supplement: Supplementary Information — Supplementary Figures, Supplementary Tables, Supplementary Discussion and Supplementary References [file ncomms15455-s1.pdf]

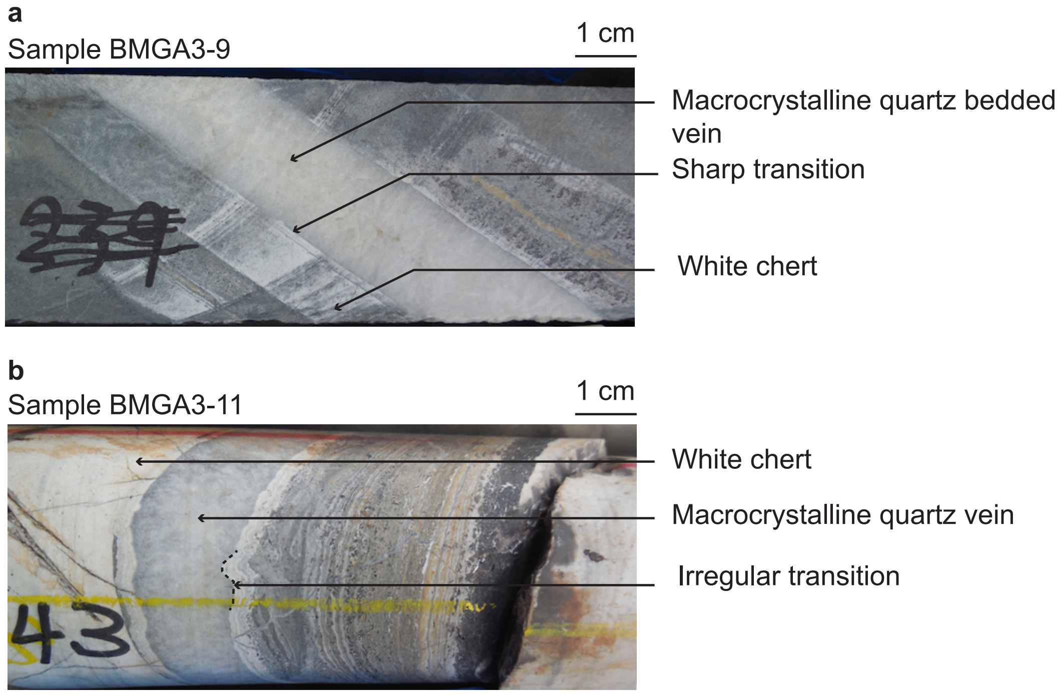

Supplementary Figure 1: Photographs of two samples from the BARB3 core. (a) Sample BMGA3-9 is a vein intruded in white chert and filled with macro-crystalline quartz crystals. The contact between the two lithologies is sharp and planar. (b) Sample BMGA3-11 is a macro-crystalline quartz vein intruded in a lithology dominated by white chert and alternating black and white cherts. The contact between the vein and the intruded rock is sharp and irregular.

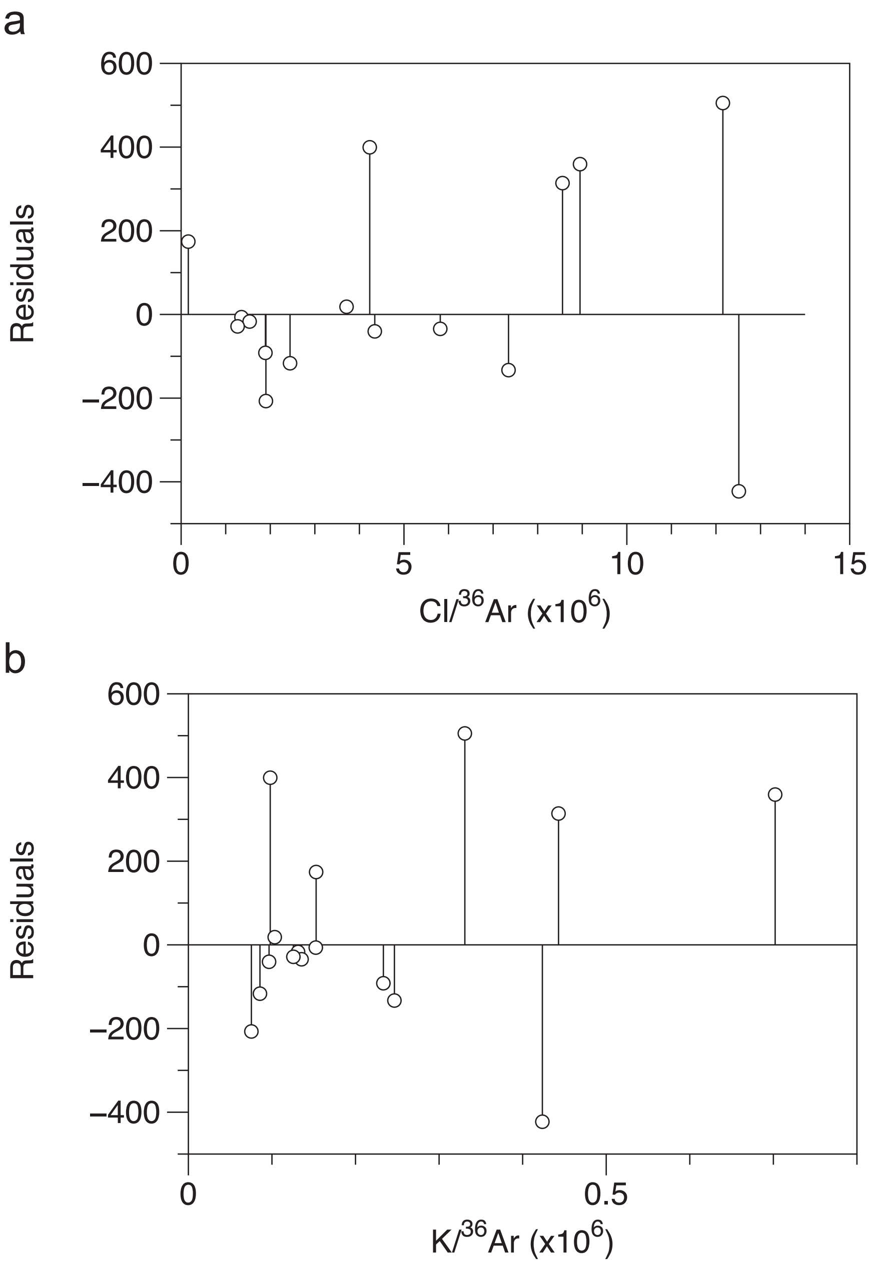

Supplementary Figure 2: Plots of residuals. (a) Residuals vs. Cl/^{36}Ar values. (b) Residuals vs. K/^{36}Ar values.

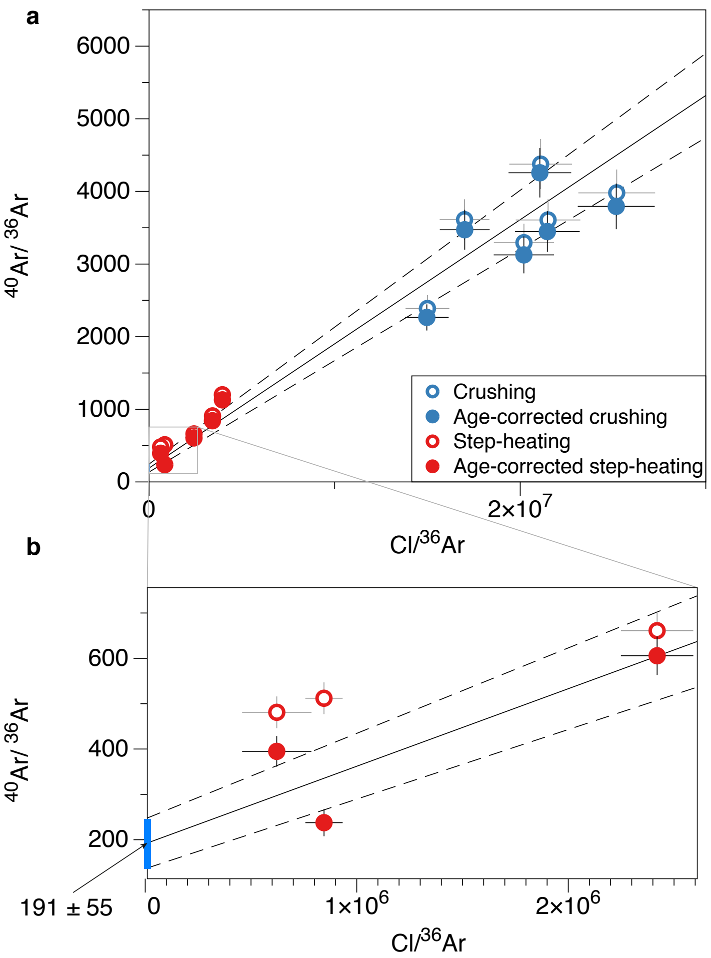

Supplementary Figure 3: Plot of $^{40}Ar/^{36}Ar$ vs. $Cl/^{36}Ar$ values for crushing (blue) and step-heating (red) experiments on sample BMGA3-13. Effect of correction for an age of 3.3 Ga is shown with the filled circles. (a) Data aligned along a correlation representing mixing between atmospheric (low $^{40}Ar/^{36}Ar$ ratios and chlorine content) and hydrothermal (high ^{40}Ar and chlorine content) components. (b) Insert zoom showing the intercept (blue range) of the line with the y-axis corresponding here to an initial $^{40}Ar/^{36}Ar$ of 191 ± 55 (1σ, s.d.).

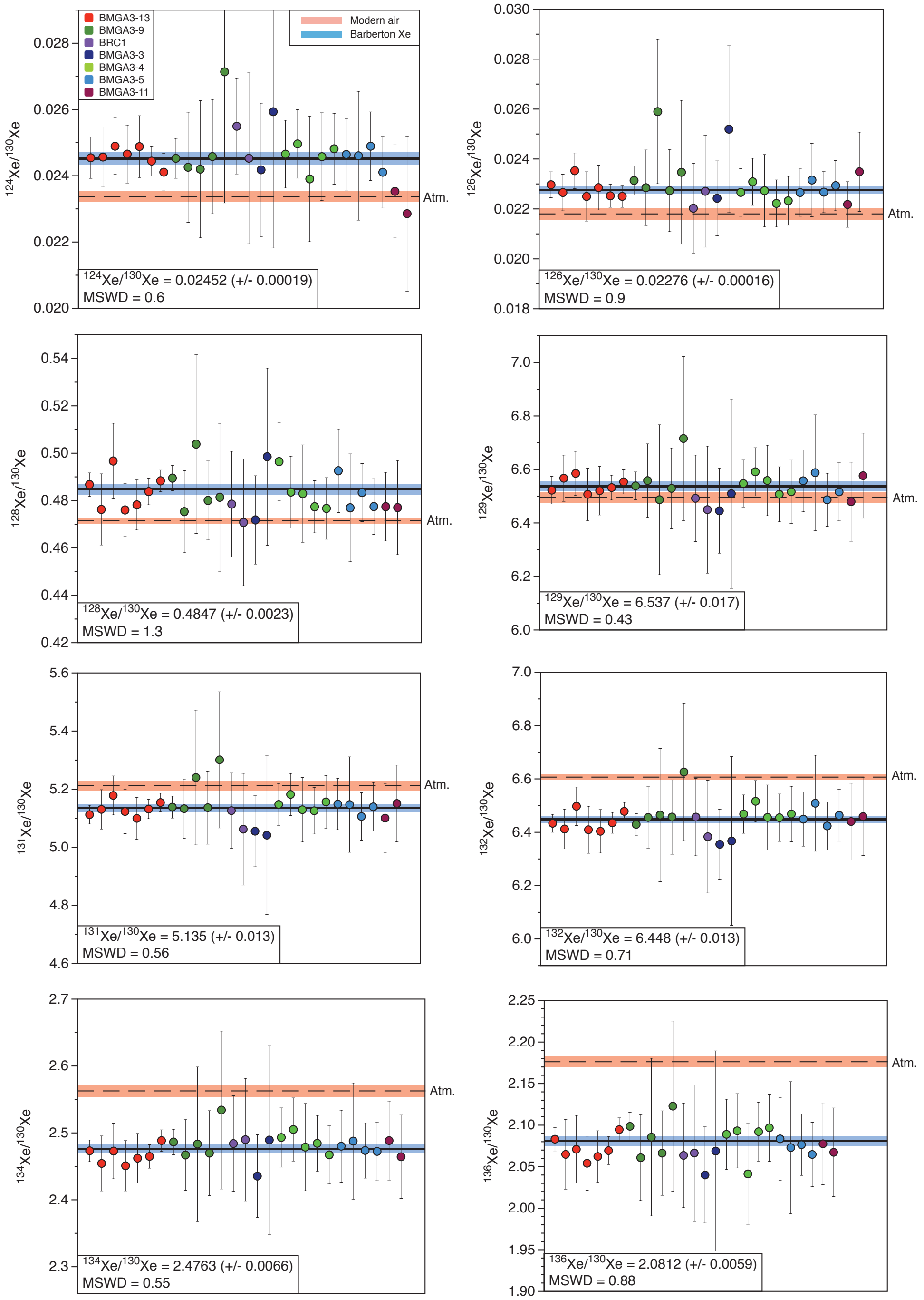

Supplementary Figure 4: Isotopic ratios (27 measurements) of xenon (normalized to ^{130}Xe) released from fluid inclusions by crushing 7 different Barberton samples. The error-weighted mean for the xenon in Barberton is shown as a solid black line and its error envelope is the blue area. The isotopic composition of modern air (dashed line) and its precision (orange area) are shown for comparison. Error bars and ranges at 2σ.

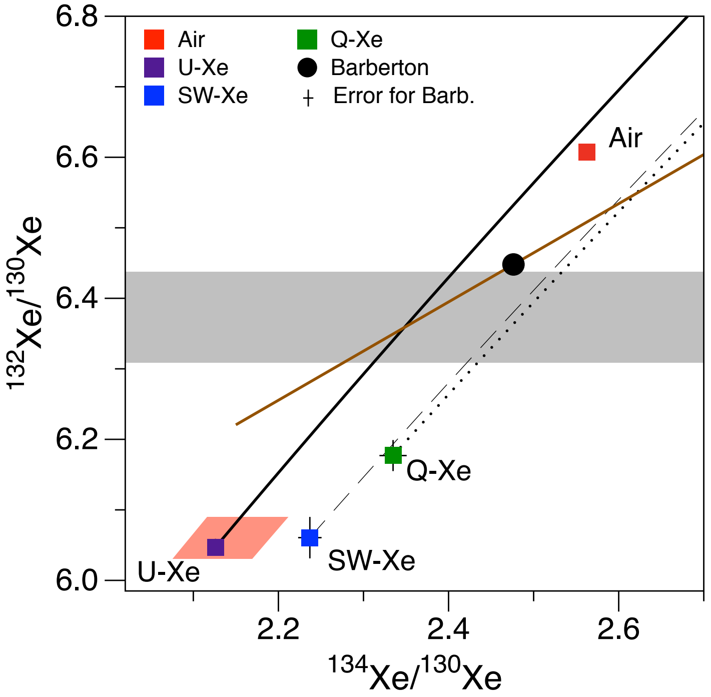

Supplementary Figure 5: Three-isotope plot $^{132}Xe/^{130}Xe$ vs. $^{134}Xe/^{130}Xe$ similar to Fig. 3. Legend is the same as in Fig. 3. A starting isotopic composition depleted in ^{134}Xe is necessary to explain results obtained for Barberton quartz samples. Errors at 2σ.

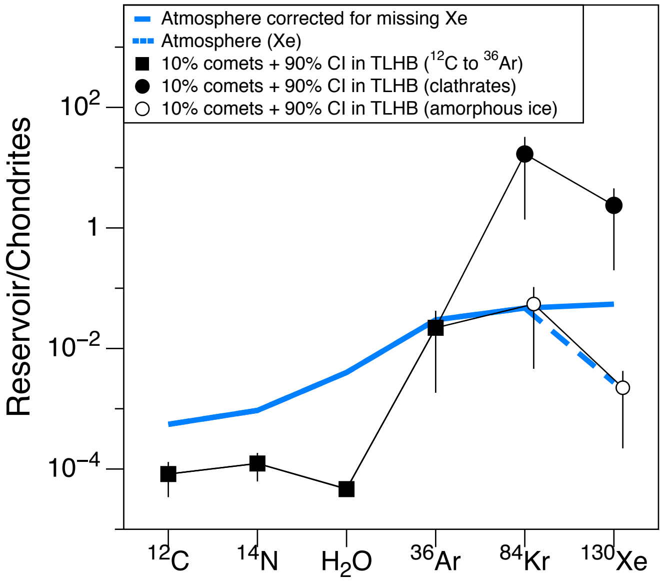

Supplementary Figure 6: Abundances of C, N, H_2O, ^{36}Ar, ^{84}Kr and ^{130}Xe in the atmosphere (air + oceans + sediments) (blue line for C, N, H_2O, ^{36}Ar, ^{84}Kr and blue dashed line for ^{130}Xe), normalized to chondritic abundances and corrected for Xe loss (blue solid line) (data from Table S4) and compared to the effect of a contribution of a Terrestrial Late Heavy Bombardment (TLHB) (2 x 10^{23} g) composed of 10% comets together with 90% CI chondrites. Atmospheric Xe partly of cometary origin cannot be excluded and is highly dependent on the mode of entrapment of Xe in comets (in clathrates or in amorphous ice).

Supplementary Table 1: Data for the Ar-Ar experiment (crushing and step-heating results) on BMGA3-9, BMGA3-13 and BMGA3-3 samples. Errors at 1σ. ASW is for Air Saturated Water.

	^{36}Ar ($x10^{-14}$ $mol.g^{-1}$)	±	$^{40}Ar/^{36}Ar$	±	$Cl/^{36}Ar$ ($x10^{6}$)	±	$K/^{36}Ar$ ($x10^{6}$)	±	Age (Ga)	±
**Crushing**										
BMGA3-9 C#1	2.05	0.13	5809	479	8.54	0.73	n.d.		n.d.	
BMGA3-9 C#2	4.43	0.25	3207	250	5.39	0.43	n.d.		n.d.	
BMGA3-9 C#3	5.02	0.28	4050	314	7.35	0.59	2.01	0.25	9.08	0.17
BMGA3-9 C#4	0.44	0.07	5479	875	8.95	1.45	4.36	0.70	8.28	0.04
BMGA3-9 C#5	1.74	0.13	5142	470	8.56	0.80	2.83	0.44	8.93	0.23
BMGA3-9 C#6	1.31	0.08	7077	571	12.15	1.02	2.56	0.33	9.71	0.18
BMGA3-9 C#7	0.81	0.05	6409	520	12.51	1.04	3.87	0.42	8.78	0.13
**Step-heating A**										
BMGA3-9 HA-600	9.58	0.45	2991	197	4.23	0.30	0.97	0.10	9.80	0.14
BMGA3-9 HA-1000	4.17	0.21	1236	84	1.35	0.14	1.88	0.15	6.74	0.08
BMGA3-9 HA-1200	4.16	0.23	757	55	0.16	0.26	0.72	0.15	7.19	0.36
BMGA3-9 HA-1400	8.39	0.39	2365	156	3.71	0.26	1.13	0.10	9.05	0.11
BMGA3-9 HA-1500	6.94	0.34	1212	82	1.54	0.12	0.30	0.13	9.99	0.79
BMGA3-9 HA-1600	9.39	0.48	1222	84	1.90	0.14	0.62	0.08	8.68	0.18
**Step-heating B**										
BMGA3-9 HB-600	9.66	0.46	3326	221	5.81	0.40	0.89	0.14	10.16	0.25
BMGA3-9 HB-1000	4.07	0.21	1441	100	1.89	0.18	2.31	0.23	6.72	0.13
BMGA3-9 HB-1200	3.55	0.19	1095	77	1.27	0.14	0.70	0.13	8.19	0.29
BMGA3-9 HB-1400	7.98	0.39	2617	176	4.34	0.30	1.13	0.10	9.25	0.09
BMGA3-9 HB-1600	10.83	0.52	1589	106	2.44	0.17	0.80	0.09	8.83	0.15
**Crushing**										
BMGA3-13 C#1	7.96	0.44	3611	57	17.01	1.34	2.17	0.20	8.72	0.08
BMGA3-13 C#2	3.72	0.21	4376	90	21.06	1.69	1.87	0.18	9.36	0.09
BMGA3-13 C#3	1.51	0.09	3607	83	21.46	1.74	2.50	0.27	8.47	0.13
BMGA3-13 C#4	1.10	0.07	3980	111	25.17	2.07	2.91	0.31	8.39	0.12
BMGA3-13 C#5	1.27	0.07	3294	72	20.20	1.62	2.62	0.31	8.20	0.16
BMGA3-13 C#6	2.83	0.16	2388	33	14.97	1.18	1.92	0.16	8.12	0.07
**Step-heating**										
BMGA3-13 600	6.84	0.33	1200	16	3.95	0.28	1.10	0.09	7.62	0.09
BMGA3-13 1000	4.85	0.25	512	11	0.85	0.09	4.30	0.30	2.95	0.05
BMGA3-13 1200	2.77	0.16	481	16	0.62	0.16	1.35	0.20	4.52	0.23
BMGA3-13 1400	6.02	0.29	909	12	3.42	0.24	1.04	0.08	7.03	0.08
BMGA3-13 1600	9.50	0.45	661	7	2.42	0.17	0.86	0.08	6.45	0.11
**Crushing**										
BMGA3-3 C#1	0.65	0.04	6179	513	19.07	1.61	3.43	0.43	8.93	0.17
BMGA3-3 C#2	0.76	0.05	7326	605	14.25	1.20	4.16	0.45	8.90	0.13
BMGA3-3 C#3	0.30	0.05	5657	1041	9.83	1.97	5.04	1.07	8.08	0.19
BMGA3-3 C#4	0.70	0.08	7131	914	14.18	1.85	4.52	0.69	8.71	0.15
BMGA3-3 C#5	0.32	0.04	7173	1074	15.97	2.42	5.55	1.24	8.35	0.30
BMGA3-3 C#6	0.68	0.07	8558	943	17.36	1.93	4.30	0.63	9.13	0.18
**Step-heating**										
BMGA3-3 600	5.69	0.29	2303	159	3.25	0.23	2.74	0.20	7.41	0.04
BMGA3-3 1000	2.85	0.20	1317	111	1.29	0.19	13.70	1.17	3.53	0.03
BMGA3-3 1200	2.62	0.15	832	61	1.07	0.17	3.58	0.36	4.66	0.12
BMGA3-3 1400	5.06	0.28	1917	140	2.53	0.44	4.85	0.43	6.03	0.09
BMGA3-3 1600	8.17	0.39	1138	76	1.32	0.12	2.65	0.20	5.94	0.06
ASW			298.56		10-17					

Supplementary Table 2: Isotopic ratios and abundances of Xe released from fluid inclusions during crushing experiments. Errors at 2σ.

Barberton samples[1]	Location (m)	^{130}Xe ($x10^{-17}$ mol.g^{-1})	±	^{124}Xe	±	^{126}Xe	±	^{128}Xe	±	^{129}Xe	±	^{131}Xe	±	^{132}Xe	±	^{134}Xe	±	^{136}Xe	±
				^{130}Xe = 1															
BMGA3-13 A	555	17.44	0.16	0.0245	0.0006	0.0230	0.0005	0.4868	0.0049	6.52	0.05	5.11	0.03	6.43	0.03	2.47	0.02	2.08	0.01
BMGA3-13 B	-	21.85	0.34	0.0246	0.0009	0.0227	0.0007	0.4763	0.0150	6.57	0.09	5.13	0.07	6.41	0.07	2.45	0.04	2.06	0.04
BMGA3-13 C	-	16.05	0.24	0.0249	0.0009	0.0235	0.0007	0.4967	0.0160	6.59	0.08	5.18	0.07	6.50	0.07	2.47	0.04	2.07	0.04
BMGA3-13 D	-	14.41	0.17	0.0247	0.0009	0.0225	0.0010	0.4760	0.0112	6.51	0.10	5.12	0.08	6.41	0.09	2.45	0.04	2.05	0.03
BMGA3-13 E	-	19.18	0.21	0.0249	0.0009	0.0229	0.0009	0.4782	0.0106	6.52	0.09	5.10	0.07	6.40	0.08	2.46	0.04	2.06	0.03
BMGA3-13 F	-	15.91	0.20	0.0244	0.0005	0.0225	0.0005	0.4838	0.0056	6.53	0.05	5.13	0.03	6.44	0.04	2.46	0.02	2.07	0.02
BMGA3-13 G	-	17.10	0.16	0.0241	0.0006	0.0225	0.0004	0.4883	0.0045	6.55	0.05	5.15	0.03	6.48	0.03	2.49	0.02	2.09	0.01
BMGA3-9 A	238	6.32	0.06	0.0245	0.0006	0.0231	0.0006	0.4895	0.0053	6.54	0.05	5.14	0.04	6.43	0.04	2.49	0.02	2.10	0.02
BMGA3-9 B	-	3.38	0.08	0.0243	0.0017	0.0228	0.0015	0.4753	0.0173	6.56	0.14	5.13	0.10	6.46	0.12	2.47	0.05	2.06	0.05
BMGA3-9 B2	-	0.84	0.03	0.0242	0.0021	0.0259	0.0029	0.5038	0.0377	6.49	0.28	5.24	0.23	6.46	0.25	2.48	0.12	2.09	0.09
BMGA3-9 C	-	3.77	0.09	0.0246	0.0017	0.0227	0.0016	0.4801	0.0167	6.53	0.15	5.14	0.13	6.46	0.14	2.47	0.06	2.07	0.05
BMGA3-9 C2	-	0.94	0.03	0.0271	0.0040	0.0235	0.0029	0.4814	0.0312	6.72	0.31	5.30	0.23	6.63	0.26	2.53	0.12	2.12	0.10
BRC B	outcrop	2.54	0.10	0.0255	0.0014	0.0220	0.0018	0.4785	0.0223	6.49	0.16	5.13	0.13	6.46	0.15	2.48	0.07	2.06	0.06
BRC B2	-	2.08	0.05	0.0245	0.0026	0.0227	0.0022	0.4708	0.0267	6.45	0.24	5.06	0.19	6.38	0.21	2.49	0.09	2.07	0.08
BMGA3-3 A	717	2.48	0.06	0.0242	0.0020	0.0224	0.0015	0.4718	0.0187	6.45	0.16	5.06	0.12	6.35	0.13	2.44	0.06	2.04	0.06
BMGA3-3 A2	-	0.60	0.03	0.0259	0.0041	0.0252	0.0033	0.4985	0.0374	6.51	0.35	5.04	0.27	6.37	0.32	2.49	0.14	2.07	0.12
BMGA3-4 A	715	12.52	0.22	0.0247	0.0010	0.0227	0.0010	0.4965	0.0166	6.55	0.09	5.15	0.07	6.47	0.07	2.49	0.04	2.09	0.04
BMGA3-4 B	-	14.00	0.21	0.0250	0.0010	0.0231	0.0009	0.4836	0.0151	6.59	0.09	5.18	0.07	6.52	0.08	2.51	0.05	2.09	0.04
BMGA3-4 B2	-	2.60	0.09	0.0239	0.0019	0.0227	0.0014	0.4829	0.0205	6.56	0.13	5.13	0.11	6.46	0.12	2.48	0.07	2.04	0.06
BMGA3-4 C	-	9.53	0.13	0.0246	0.0013	0.0222	0.0009	0.4774	0.0110	6.51	0.10	5.13	0.08	6.45	0.09	2.48	0.04	2.09	0.04
BMGA3-4 D	-	8.23	0.12	0.0248	0.0011	0.0223	0.0010	0.4767	0.0130	6.52	0.12	5.16	0.09	6.47	0.10	2.47	0.04	2.10	0.04
BMGA3-5 A	784	11.02	0.22	0.0246	0.0011	0.0227	0.0009	0.4926	0.0176	6.56	0.12	5.15	0.09	6.45	0.10	2.48	0.05	2.08	0.05
BMGA3-5 A2	-	2.37	0.16	0.0246	0.0019	0.0232	0.0015	0.4770	0.0228	6.59	0.22	5.15	0.16	6.51	0.18	2.49	0.09	2.07	0.08
BMGA3-5 B	-	10.70	0.14	0.0249	0.0010	0.0227	0.0008	0.4834	0.0123	6.49	0.10	5.11	0.08	6.42	0.09	2.47	0.04	2.08	0.04
BMGA3-5 C	-	15.19	0.24	0.0241	0.0011	0.0229	0.0010	0.4775	0.0118	6.52	0.11	5.14	0.08	6.46	0.10	2.47	0.04	2.06	0.04
BMGA3-11 A	843	8.05	0.18	0.0235	0.0014	0.0222	0.0009	0.4774	0.0145	6.48	0.15	5.10	0.12	6.44	0.14	2.49	0.06	2.08	0.05
BMGA3-11 A2	-	2.14	0.05	0.0229	0.0023	0.0235	0.0016	0.4770	0.0199	6.58	0.16	5.15	0.13	6.46	0.15	2.46	0.06	2.07	0.05
BMGA3-12 A	-	0.31	0.02	0.0253	0.0050	0.0230	0.0046	0.4299	0.0518	6.47	0.54	5.03	0.43	6.39	0.50	2.45	0.22	2.03	0.20
**Average**				**0.0245**	**0.0002**	**0.0228**	**0.0002**	**0.4847**	**0.0023**	**6.537**	**0.017**	**5.135**	**0.013**	**6.448**	**0.014**	**2.476**	**0.007**	**2.081**	**0.006**
MSWD*				0.55		0.87		1.3		0.43		0.56		0.71		0.55		0.88	

[1]Barberton samples are labeled BMGA3-XX-YZ with XX the core samples, Y the 1-3 mm granulometric sub fraction and Z the crushing step excepted the "BRC" sample from one outcrop

*MSWD (for Mean Standard Weighted Deviation)

Supplementary Table 3: Isotopic ratios of krypton released from fluid inclusions during crushing experiments on Barberton quartz samples. Errors at 2σ.

Samples	Location (m)	^{80}Kr	±	^{82}Kr	±	^{83}Kr	±	^{86}Kr	±
		^{84}Kr=1							
BMGA3-13-G	555	0.0396	0.0004	0.2021	0.0018	0.2013	0.0016	0.3074	0.0017
BMGA3-13 B	555			0.2031	0.0015	0.2021	0.0013	0.3056	0.0025
BMGA3-13 C	-			0.2020	0.0017	0.2019	0.0016	0.3020	0.0027
BMGA3-13 D	-			0.2031	0.0015	0.2021	0.0013	0.3056	0.0025
BMGA3-13 E	-			0.2022	0.0009	0.2012	0.0007	0.3059	0.0028
BMGA3-4 A	715			0.1992	0.0021	0.1979	0.0024	0.3015	0.0041
BMGA3-4 B	-			0.2019	0.0020	0.2018	0.0015	0.3042	0.0024
BMGA3-4 B2	-			0.2021	0.0091	0.1995	0.0083	0.3034	0.0132
BMGA3-4 C	-			0.2023	0.0078	0.2016	0.0016	0.3042	0.0035
BMGA3-5 A	784			0.2017	0.0025	0.2015	0.0025	0.3028	0.0032
BMGA3-5 B	-			0.2012	0.0063	0.2015	0.0055	0.3058	0.0105
BMGA3-5 C	-			0.2024	0.0018	0.2021	0.0013	0.3044	0.0030
**Average**				**0.20217**	**0.0005**	**0.20151**	**0.00042**	**0.30499**	**0.00082**
MSWD*				1.05		1.2		1.7	

[1]Barberton samples are labeled BMGA3-XX-YZ with XX the core samples, Y the 1-3 mm granulometric sub fraction and Z the crushing step.
*MSWD (for Mean Square Weighted Deviation)

Supplementary Table 4: Abundances of volatile elements in chondrites (after a compilation of results, ref. 1-3**), in the atmosphere (air + oceans + sediments, ref.** 4**) and in bulk comets composed of clathrates or amorphous ice (see text for details) all normalized to chondritic abundances.** Note the differences in ^{84}Kr and ^{130}Xe abundances between clathrates and amorphous ices. Errors at 1σ.

Abundances (mol g^{-1} for Chondrites)	Chondrites		Atmosphere / Chondrites		Comets bulk (clathrate) / Chondrites		Comets bulk (amorphous ice) / Chondrites	
^{12}C	2.34E-03	1.17E-03	5.51E-04	2.75E-04	1.58E+01	1.45E+01	1.58E+01	1.45E+01
^{14}N	6.38E-05	2.48E-05	9.38E-04	3.65E-04	2.81E+01	1.84E+01	2.81E+01	1.84E+01
H_2O	3.70E-03	1.02E-03	4.00E-03	1.10E-03	4.91E+00	2.70E-01	4.91E+00	2.70E-01
^{36}Ar	3.13E-11	1.60E-11	2.98E-02	1.52E-02	6.60E+03	6.05E+03	6.60E+03	6.05E+03
^{84}Kr	4.09E-13	2.22E-13	4.68E-02	2.54E-02	5.06E+06	4.64E+06	1.64E+04	1.50E+04
^{130}Xe	3.84E-14	3.15E-14	2.72E-03	2.24E-03	7.10E+05	6.51E+05	6.57E+02	6.02E+02

Supplementary Table 5: Elemental ratios of noble gases (Ar, Kr, Xe) in cometary ices (amorphous ice or clathrates). Starting solar abundances are from ref. 5. Data for amorphous ice from ref. 6 and for clathrates from ref. 7.

Elemental ratio	Protosolar nebula	Amorphous ice	Clathrates
$^{36}Ar/^{130}Xe$	3.28E+05	8.20E+03	7.57E+00
$^{36}Ar/^{84}Kr$	2.47E+03	3.09E+01	9.98E-02

Supplementary Discussion

In Barberton quartz, ^{124}Xe appears depleted relative to the fractionation experienced by neighboring light isotopes of Xe, and for this reason it has been excluded when calculating the extent of mass dependent fractionation. This mono-isotopic depletion remains hard to explain since there are relatively few processes (radioactive decay or neutron capture) able to selectively decrease the abundance of this isotope. Radioactive decay of ^{124}Xe may occur by double electron capture. However the half-life ($T_{1/2}$) of ^{124}Xe is higher than 4.10^{20} a (ref. 8) and there is no reason to expect that this process occurred only in-situ and not in all cosmochemical or terrestrial reservoirs including the terrestrial atmosphere that is used here to normalize isotopic spectra. Thermal neutron capture on ^{124}Xe leads to the production of ^{125}Xe decaying rapidly ($T_{1/2}$ = 18 hr) to ^{125}I that is itself unstable decaying ($T_{1/2}$ = 60 days) to ^{125}Te (ref. 9). Because the modern atmosphere, after correction for mass-dependent isotopic fractionation, does not show a comparable depletion in ^{124}Xe relative to potential primordial components, neutron capture might have taken place in-situ. The rate of depletion in ^{124}Xe depends on both the thermal neutron capture cross-section of ^{124}Xe and on the crustal neutron flux. Interestingly, the thermal neutron capture cross-section of ^{124}Xe is high (165 ± 11 barns) compared to other Xe isotopes (0.26 to 85 barns, ref. 9**)**. Following the rationale presented in ref. 10, the depletion in ^{124}Xe by thermal neutron capture is given by equation (1):

$$^{124}Xe_{nc} = P_{th} \times N \times F_{^{124}Xe} \qquad (1)$$

where P_{th} is the probability for neutrons to be thermalized (≈ 0.8), N is the neutron flux in the crust (11.2 neutrons g^{-1} a^{-1}), and F_{124Xe} is the probability that ^{124}Xe will capture a thermal neutron. This corresponds to the neutron capture probability of ^{124}Xe (abundance in quartz ≈ 10^{-16} mol g^{-1}, Supplementary Table 2) multiplied by the thermal neutron capture

cross-section (165 barns)) divided by the total neutron capture probability in the crust (0.00979 mol barns). All values, except the thermal neutron capture cross-section of ^{124}Xe, are from ref. 10. Calculations lead to a rate of removal of ^{124}Xe atoms by thermal neutron capture of 7.10^{-37} mol g^{-1} a^{-1} corresponding to only 2.10^{-27} mol g^{-1} of ^{124}Xe integrated over 3.3 Ga (time of fluid-entrapment). This first-order estimate demonstrates that thermal neutron capture on ^{124}Xe is unlikely to be the process responsible for the depletion in ^{124}Xe recorded in Barberton quartz samples. Alternatively, the depletion in ^{124}Xe of about 20 ‰ compared to the isotopic fractionation measured in Barberton samples may be explained by a corresponding enrichment in the present atmosphere by addition of cosmogenic ^{124}Xe produced in the continental crust during the last 3.2 Ga and subsequently degassed. In this case the main process responsible for production of cosmogenic Xe isotopes would be by spallation of Ba by neutrons. However, this nuclear reaction produces both ^{126}Xe and ^{124}Xe isotopes with a ^{126}Xe/^{124}Xe value of about 1.7 (ref. 11). This elevated ratio is not consistent with the atmospheric ^{126}Xe/^{124}Xe value and demonstrates that cosmogenic production during 3.3 Ga is unlikely to explain the depletion in ^{124}Xe in Archean quartz, since the neutron capture would have also produced ^{126}Xe excesses in the atmosphere that are not observed.

The depletion of ^{134,136}Xe isotopes in the Barberton Xe spectrum (Fig. 5) demonstrates that neither Q-Xe nor SW-Xe can be the starting isotopic compositions for the Earth's atmosphere (see main text). Fractionating Q-Xe or SW-Xe would lead to excesses in ^{134}Xe and ^{136}Xe compared to Barberton. Once corrected for a mass fractionation of 25 ‰.u^{-1} relative to U-Xe, $^{131-136}$Xe excesses in Barberton quartz are fully compatible with the presence of products of the spontaneous fission of ^{238}U (Fig. 9).

Estimates of cometary bulk compositions containing amorphous ice or clathrates are listed in Supplementary Table 4. Carbon, Nitrogen, water and ^{36}Ar contents are from estimates by Marty et al. (ref. 12) and do not depend on the modes of formation of ice

(clathrates or amorphous ice) however ^{84}Kr and ^{130}Xe abundances highly depend on the structure of cometary ice. Here we used experimental data obtained during amorphous ice deposition[6,13] and theoretical data on the elemental composition of clathrates formed at 48 K (ref. 7) to give estimates of the ^{36}Ar/^{130}Xe and ^{36}Ar/^{84}Kr (Supplementary Table 5) trapped in cometary amorphous ices or clathrates formed from a gas with a protosolar composition[5]. The ^{36}Ar/H_2O ratio is from the measurement of gases emitted by comet 67P/C-G by the ROSINA mass spectrometer on board the Rosetta spacecraft[14] and the H_2O content is from ref. 15. Nitrogen and carbon contents are from a compilation of published data[16,17]. In this study, we explored the effect of a cometary contribution of 10 % wt. in the Terrestrial Late Heavy Bombardment (TLHB) and used a total mass of 2 x 10^{23} g for this late accretionary event[12,18]. The Late Heavy Bombardment is taken here as an example only. The data presented in this study do not provide constraints on the accretion event responsible for delivering the budget of atmospheric Xe. During such an event, the contribution to the budget of volatile elements from the remaining 90 % wt. of chondrites is negligible[18]. The effect on C, N and H_2O abundances is small (Supplementary Fig. 6) and ensures, for example, a preservation of the chondritic D/H value for Earth's oceans despite the elevated cometary D/H measured for comets (*e.g.* ref. 19). In contrast to C, N and H_2O, the effect of a 10 % cometary contribution in the TLHB is significant for ^{36}Ar, ^{84}Kr and ^{130}Xe abundances (Supplementary Fig. 6). Comets may deliver the entire surficial budget of ^{36}Ar. The cometary contribution to the TLHB is sufficient to account for the atmospheric ^{84}Kr budget if it was trapped in amorphous ice, however this would result in a cometary ^{130}Xe abundance being equal to the actual budget in the Earth's atmosphere uncorrected for the 20 fold depletion[20]. If noble gases were trapped in clathrates, a 10% cometary contribution would exceed by 2 to 3 orders of magnitude the surficial budget of Xe and Kr. In summary, a cometary contribution to the Earth's is tentative for ^{36}Ar and cannot be ruled out for ^{84}Kr and ^{130}Xe.

Supplementary References

1. Schultz, L. & Franke, L. Helium, neon, and argon in meteorites: A data collection. *Meteorit Planet Sci* **39,** 1889–1890 (2004).
2. Kerridge, J. F. Carbon, hydrogen and nitrogen in carbonaceous chondrites: Abundances and isotopic compositions in bulk samples. *Geochimica et Cosmochimica Acta* **49,** 1707–1714 (1985).
3. Mazor, E., Heymann, D. & Anders, E. Noble gases in carbonaceous chondrites. *Geochimica et Cosmochimica Acta* **34,** 781–824 (1970).
4. Marty, B. The origins and concentrations of water, carbon, nitrogen and noble gases on Earth. *EPSL* **313-314,** 56–66 (2012).
5. Lodders, K. Solar system abundances and condensation temperatures of the elements. *ApJ* **591,** 1220 (2003).
6. Notesco, G. Gas trapping in water ice at very low deposition rates and implications for comets. *Icarus* **162,** 183–189 (2003).
7. Mousis, O., Lunine, J. I., Picaud, S. & Cordier, D. Volatile inventories in clathrate hydrates formed in the primordial nebula. *Faraday Discuss.* **147,** 509–525 (2010).
8. Suhonen, J. Double beta decays of 124Xe investigated in the QRPA framework. *J. Phys. G: Nucl. Part. Phys.* **40,** 1–14 (2013).
9. Bresesti, M., Cappellani, F., Del Turco, A. M. & Orvini, E. The thermal neutron capture cross-section and the resonance capture integral of 124Xe. *J. Inorg. Nucl. Chem.* **26,** 9–14 (1964).
10. Ballentine, C. J. & Burnard, P. G. Production, Release and Transport of Noble Gases in the Continental Crust. *RiMG* **47,** 481–538 (2002).
11. Kaiser, W. A. The Excitation Functions of Ba (p, X) MXe (M= 124-136) in the Energy Range 38-600 MeV; The Use of 'Cosmogenic' Xenon for Estimating 'Burial' Depths and ‘Real’ Exposure Ages. *PTRSA* **285,** 337–362 (1977).
12. Marty, B. *et al.* Origins of volatile elements (H, C, N, noble gases) on Earth and Mars in light of recent results from the ROSETTA cometary mission. *EPSL* **441,** 91–102 (2016).
13. Bar-Nun, A. & Owen, T. in *Solar System Ices* (ed. Schmitt, B.) **227,** 353–366 (Springer Netherlands, 1998).
14. Balsiger, H. *et al.* Detection of argon in the coma of comet 67P/Churyumov-Gerasimenko. *Science Advances* **1,** 1–4 (2015).
15. Greenberg, J. M. Making a comet nucleus. *A&A* **330,** 375–380 (1998).
16. Le Roy, L., Altwegg, K., Balsiger, H. & Berthelier, J. J. The inventory of the volatiles on comet 67P/Churyumov-Gerasimenko from Rosetta/ROSINA. *A & A* (2015).
17. Rubin, M., Altwegg, K., Balsiger, H. & Bar-Nun, A. Molecular nitrogen in comet 67P/Churyumov-Gerasimenko indicates a low formation temperature. *Science* (2015). doi:10.1126/science.aaa0193
18. Marty, B. & Meibom, A. Noble gas signature of the late heavy bombardment in the Earth's atmosphere. *EEarth* **2,** 43–49 (2007).
19. Altwegg, K. *et al.* 67P/Churyumov-Gerasimenko, a Jupiter family comet with a high D/H ratio. *Science* **347,** 1–3 (2015).
20. Avice, G. & Marty, B. The iodine-plutonium-xenon age of the Moon-Earth system revisited. *Phil. Trans. R. Soc. A* **372,** 1–16 (2014).
